# Reciprocity in global health: How successes from the global south can inform global health interventions in Canada and the U.S

**DOI:** 10.1371/journal.pgph.0007208

**Published:** 2026-08-27

**Authors:** Tsitsi B. Masvawure, Mary Ndu, Charity Oga-Omenka

**Affiliations:** 1 Dept of Integrative and Global Studies, The Global School, Worcester Polytechnic Institute, Worcester, Massachusetts, United States of America; 2 School of Public Health Sciences, University of Waterloo, Ontario, Canada; Institute of Tropical Medicine: Instituut voor Tropische Geneeskunde, BELGIUM

## Abstract

Global health has traditionally functioned as a project of high-income countries (HICs) addressing health challenges in low- and middle-income countries (LMICs). This “outward facing” orientation has created conceptual and operational boundaries that obscure the persistence of health inequities within HICs and perpetuates the false assumption that resource-rich nations have resolved fundamental health equity challenges in their own countries. In this essay we argue that HICs have global health problems of their own and should be considered legitimate sites for global health intervention. We propose an approach to doing global health in the global north that is based on reciprocal learning and where solutions to global health problems can also be found outside the borders of HICs. Complementing the extant scholarship on the need to decolonize global health, this essay highlights LMIC-led successes that can inform global health practice in the U.S. and Canada thus positioning global health as a truly egalitarian project.

## Introduction

Two years ago, we thought about writing an essay on how to “do” global health in the global north, that is, how to implement global health interventions for populations based in high-income countries (HICs). We were, and still are, frustrated by the continued use of the term “global health” to refer predominantly to health issues in so-called low-to-middle income countries (LMICs) even though Koplan et al’s seminal definition explicitly states that the “global” in global health refers to the “scope of an issue”, not to geographic location [1]. We often get confused, even amused reactions when we explain to others that we actively teach about global health problems in HICs in our global health courses. Many express curiosity, but remain wary: will students sign up for a global health course that is not exclusively about problems “over there” (i.e., in LMICs)? Indeed, many students taking our global health courses expect to learn about health challenges in distant, resource-limited settings. This reflects how deeply entrenched this “outward-facing” [2] framing of global health is in the field. However, given a rapidly changing global health landscape in which the U.S. and Canada—where we are based—are looking increasingly inward and prioritizing the health and wellbeing of their own populations [3,4], this essay could not be timelier.

We seek to broaden understanding of global health beyond the narrow focus on health issues in LMICs by showing that HICs also have global health challenges and should be considered legitimate sites for global health intervention. We propose an approach to doing global health in the global north that is based on reciprocal learning and where solutions to global health problems in HICs can also be found outside their borders. Reciprocal learning, also called reciprocal innovation, refers to bidirectional exchange of knowledge and challenges assumptions that innovation and expertise originate primarily in HICs and then flow to LMICs [5,6]. Reciprocal learning approaches recognise that knowledge is contextual, not universal, and thus encourage mutual learning and knowledge exchange [7,8]. This essay both complements the extant literature on the need to decolonize global health [9,10] and extends it by providing pragmatic examples of how reciprocal learning can be applied in practice to tackle global health challenges in HICs. Many papers that call for the decolonization of global health typically focus their critiques on HIC-led initiatives in the global south [9–11]. There is, however, limited scholarship that explicitly frames health issues in HICs as global health issues [12,13] or that focuses on how LMIC-led global health successes can be leveraged to help solve global health problems in HICs. We focus specifically on lessons for the U.S. and Canada in this essay.

The views we express in this essay are informed by our hybrid and dual identities as scholars from Africa who are now based in HICs—Canada and the U.S.—and who, like many other (im)migrants often exist in a liminal space: always tethered to our countries of origin even as we make new lives in our host countries. Like other (im)migrants, we have access to alternative ways, culturally, of being human and often find ourselves asking the question: “Why this approach and not that one?” Histories of colonization and neo-colonial globalization in our origin countries have made us acutely aware of race-and-class-based power inequities and inform our research. We suspect that our hybrid identities help us see opportunities to do global health in the global north that others might miss, including the possibility that HICs can learn from and be transformed by global health wisdom generated in the global south, just as LMICs have long received technical expertise and support from the global north [14]. We write, therefore, from inside the contradictions of HICs that project global health leadership abroad while reproducing structural inequity at home. This essay is organized into three sections. We begin with a brief discussion of the rapidly changing global health landscape; discuss limitations in the current framing of global health in academia, policy and practice; and present lessons from select LMIC-led successes in maternal health, tuberculosis control and community-based healthcare that can inform global health practice in HICs. The LMIC successes we present in this essay are not flawless and they have not eradicated global health challenges in the respective countries. Rather, we present these as exemplars of alternative approaches to global health that could inform global health practice elsewhere.

### A dramatically changing global health landscape

Few could have predicted the seismic changes that have occurred in the global health landscape in the past year. The sudden dismantling of USAID and drastic downsizing of other global health institutions in the U.S. have destabilized global health and thrown the industry (i.e., the network of organizations, businesses and individuals that profit from global health) [15] and academia into disarray [16,17]. For instance, nearly 300,000 people affiliated with USAID globally have lost their jobs [18], underscoring both the vulnerability and interconnectedness of Americans and non-Americans working in global health. The end of USAID has also disrupted global health partnerships between institutions in HICs and LMICs while federal funding cuts have shrunk many academic institutions’ budgets, making outward facing global health work difficult, even impossible [19]. Consequently, some academic programs in the U.S. are now seriously considering domestic global health opportunities for their students. These drastic changes to global health have also been codified at the policy level through the 2025 America First Global Health Strategy, which sets out three new priorities–making America *safer*, *stronger* and *more prosperous*–and articulates an explicitly inward-facing global health that primarily benefits America financially and technologically [20].

Canada, too, has not been immune to this inward turn. In November 2025, the Canadian government announced significant reductions to its international development and global health commitments, including cuts to Global Affairs, Canada’s international assistance envelope. While less rhetorically aggressive than the U.S. Strategy, Canada’s approach reflects a similar logic: the primary obligation of a government is to its own citizens, and global health investment must demonstrate returns at home [21]. Across the G7 and beyond, this border-conscious posture is increasingly displacing the solidarity-based framing that animated the Alma-Ata Declaration and the Millennium/Sustainable Development Goals. Simultaneously, defence and security spending are rising: Canada has committed to increasing military expenditure to meet NATO’s 2% GDP target, even as global health budgets contract [4]. This reallocation of public resources from health equity to national security reflects broader political priorities about where public investment should flow and where threats to population wellbeing are presumed to originate. Other HICs have also reduced their global health funding, with France slashing its contribution to the Global Fund by 58% [22]. Germany, the UK and Japan also reduced their overseas development aid for the first time in thirty years in 2025 [23].

However, this increasingly inward policy orientation may weaken capacities that also protect HIC populations. Pathogens do not observe funding envelopes: dismantling USAID-supported global surveillance networks in LMICs leaves critical gaps in the early warning systems that HICs depend on, a lesson underscored during the COVID-19 pandemic but one that current policy directions appear to ignore. The ongoing Ebola outbreak in the Democratic Republic of the Congo is arguably revealing the dire consequences of the America First Strategy, whose funding cuts and insularity have been blamed for contributing to a weakened global health surveillance system [24,25]. HICs also benefit disproportionately from health worker migration out of LMICs hence defunding the training partnerships and research infrastructure that produce those workers is, in effect, cutting the pipeline that staffs HIC hospitals and long-term care homes [26]. Yet, HICs simultaneously squander much of that workforce through credentialing barriers that leave internationally trained physicians and nurses in precarious employment while rural communities go without care. This imposes costs on source countries, receiving countries, and the professionals themselves [27].

**Fig 1:**
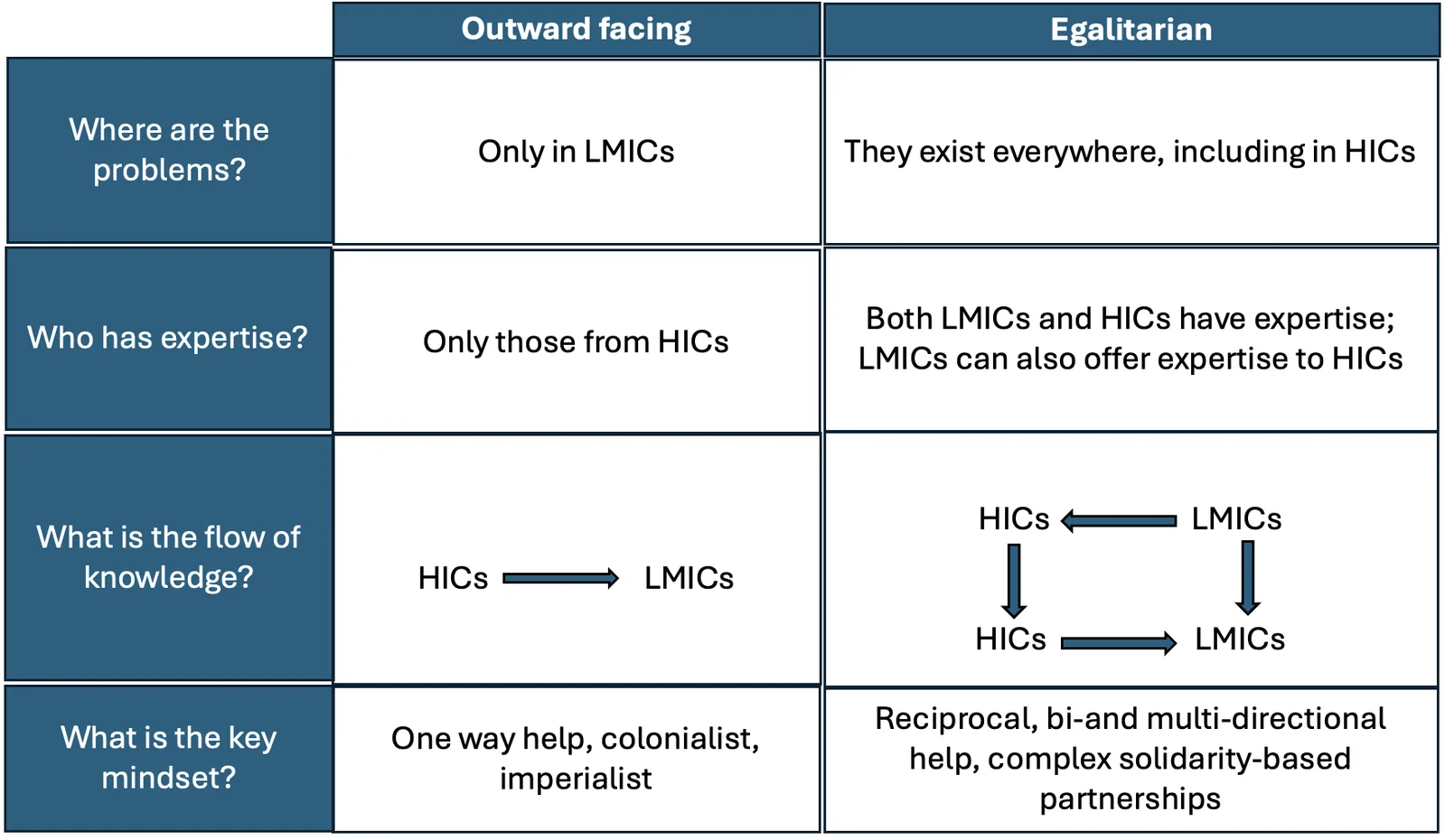
Models of knowledge flows in global health.

In making the case that HICs have global health needs, we are not advocating for insularity but are calling for genuinely egalitarian partnerships in which HICs also look to LMICs for solutions and expertise. Egalitarian global health partnerships entail bi-and multidirectional knowledge flows from LMICs to HICs and vice versa, rather than uni-directional flows from HICs to LMICs as tends to be the case [28]. In Fig 1, we show that egalitarian partnerships require a mindset shift about where knowledge and expertise reside and can help disrupt colonial legacies that unquestionably privilege western epistemologies as the only legitimate form of expertise. In this essay we focus specifically on bi-directional knowledge flows from LMICs to HICs, rather than on multi-directional knowledge flows.

### Recognising global health inequities in HICs

Global health has traditionally functioned as a project of HICs addressing health challenges in LMICs [29]. This orientation has created conceptual and operational boundaries that obscure the persistence of health inequities within HICs themselves and perpetuates the false assumption that resource-rich nations have resolved fundamental health equity challenges and thus have no “global health” problems. Despite a longstanding scholarship on health inequities in HICs [30,31], health equity as a public health priority in HICs arguably only gained traction and visibility when the concept of “social determinants of health inequalities” (SDOH) was popularised in the early 2000s [32]. The Lancet Commission on SDOH helped to galvanise attention around the need to address social inequities that fuel health inequities within HICs—and globally—rather than blaming individuals for their poor health. Consequently, most SDOH scholarship promotes health equity approaches to help tackle the profound health disparities that racialized, indigenous and other minoritized communities in HICs continue to experience despite abundant resources in these countries [33,34].

In the U.S. social inequities have seen millions, particularly low-income individuals, remain uninsured and underinsured [35] and unable to access health services or facing personal bankruptcy due to medical debt [36]. In Canada, local inequities are not abstract either. As of 2026, for instance, 41 long-term drinking water advisories remain in First Nations communities despite federal commitments made in 2015 to eliminate them [37]. The persistence of unsafe water systems reflects a longstanding infrastructure inequity rooted in colonial governance systems on reserve lands. The Canadian government has been largely unsuccessful in closing the health gap between Indigenous and non-Indigenous Canadians, despite successive Truth and Reconciliation Commission calls to action [38]. Black Canadians also carry disproportionate burdens of food insecurity, housing precarity, and poor mental health as a legacy of racialized exclusion in policy and labour markets while racialized immigrants routinely face gaps in provincial health coverage [39,40]. The growing resistance to vaccinations is yet another example of an urgent global health challenge afflicting several HICs [41]. Vaccine hesitancy contributed to America’s exceptionally high COVID-19 infection and death rates compared to several LMICs and is currently responsible for the re-emergence of measles as a major childhood disease in the country [42]. These realities show that global health is as much about internal (local) inequities as it is about international inequities and is a clear indication of shared vulnerabilities and challenges.

Many scholars have argued that the tendency to associate global health with LMICs is based on a deficit model that views other parts of the world as having nothing meaningful to contribute to disease prevention and global health solutions [11,28,29]. This deficit model also perpetuates colonialist tropes that individuals from LMICs are somehow biologically and intellectually inferior, and hence prone to disease and incapable of solving their own problems [11,28,29]. We complement these critiques by further pointing out that deficit models conveniently ignore that the U.S. and other HICs have, historically, also been sources of harmful diseases that have spread outwards to LMICs. Colonization, for instance, brought a host of previously unknown diseases to South America, the Caribbean and Africa and killed millions [43]. Even when HICs have not been sources of disease, their policies and priorities have often worsened health in LMICs, such as when the U.S. restricted access to HIV medications in Africa in the 1990s [44] or when the global gag rule regularly withholds funding for reproductive health services in LMICs [45]. Despite Canada’s reputation as a principled actor in global health, its actual funding commitments have consistently told a different story, with official development assistance remaining well below the UN target of 0.7% of GNI for over two decades [46]. Recent budget decisions have deepened this gap, cutting international assistance, including global health programming specifically. For populations that depend on Canadian-funded health programs across Africa, the Caribbean, and South Asia, these reductions represent a substantial contraction in essential health services. However, they have drawn considerably less public attention than the dismantling of USAID.

In this essay we extend critiques of global health by noting that, even when health inequities within HICs are acknowledged, we have noticed a reluctance or inability to frame these explicitly as “global health” problems unless (im)migrant populations from the global south are affected. In conversations with colleagues in the U.S., for instance, who are now having to figure out alternative global health practicums for students at their institutions in response to federal funding cuts and the changing political priorities discussed earlier, we frequently hear statements to the effect that students can get their “global health experience” without leaving the U.S. by working with (im)migrant or refugee communities from the global south. Unfortunately, this approach continues to associate global health issues with diseased bodies from the global south and highlights the difficulty many scholars, and some academic disciplines, have in applying a global health lens to HIC contexts.

Rethinking global health to be less racist and colonialist requires constantly reminding ourselves that the *global* in global health indexes a commitment to addressing health inequities wherever they exist in the world. HICs are part of the global and must turn the lens inward, albeit not in an insular sense. Acknowledging shared global vulnerabilities allows for reciprocal learning and fosters a global health that is built on solidarity and reciprocity [14] and where the knowledge and expertise of formerly colonized and/or marginalized populations is actively sought and Western expertise is not automatically assumed as the default [47]. In global health, this means that HICs must also be willing to look to LMICs for leadership and ideas for truly reciprocal and egalitarian global partnerships.

### Doing global health in HICs: Transferable lessons from LMICs

**Fig 2:**
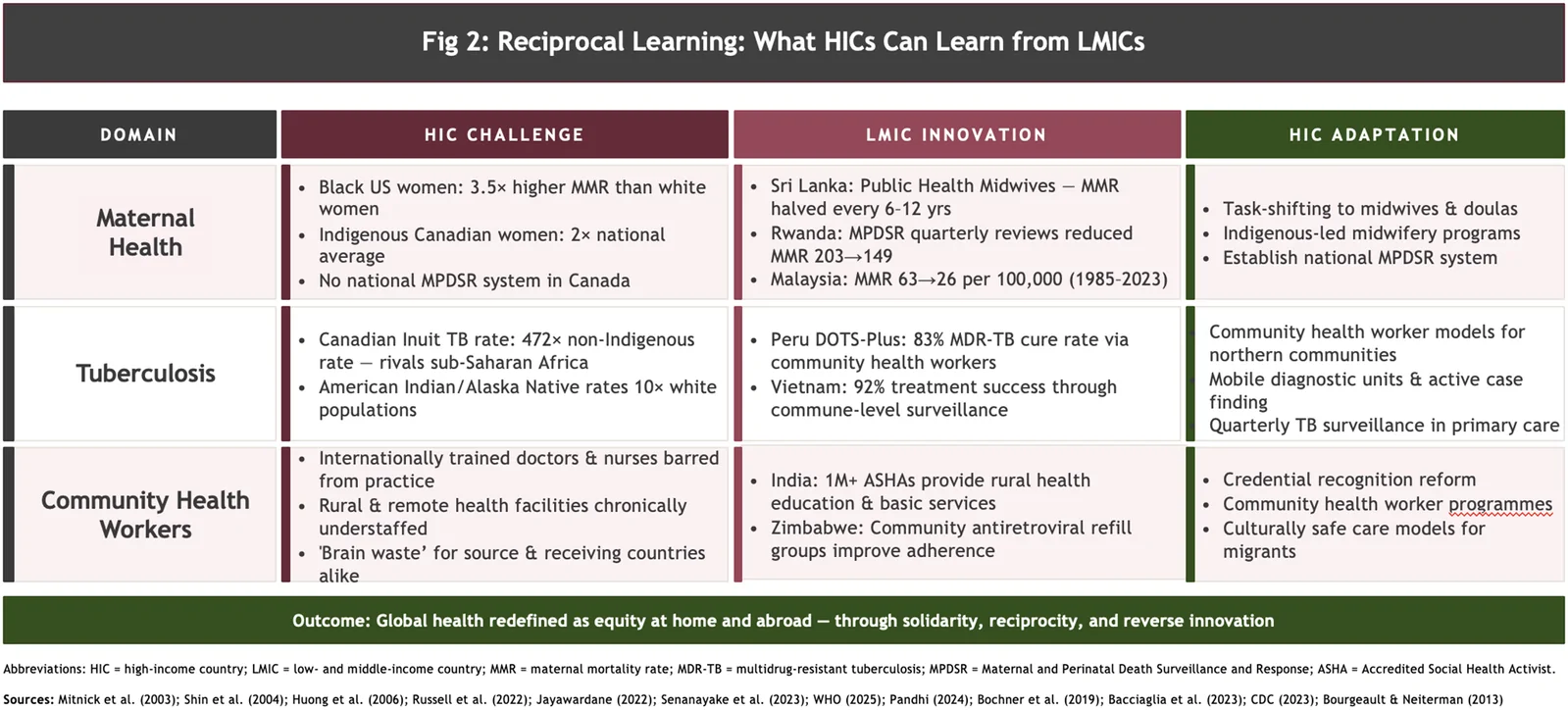
Reciprocal learning: What HICs can learn from LMICs.

In this section we present examples of global health successes and transferable strategies from LMICs in the areas of maternal health, tuberculosis and community health—which represent our areas of expertise—and propose models that can be operationalized in HICs and in the U.S. and Canada, specifically. Fig 2 summarises these successes and strategies. Our goal in this section is to highlight areas for potential reciprocal learning; it is not to suggest that nothing is being done in Canada or the U.S. to address health inequities in these areas nor is it to imply that the LMIC strategies are faultless. We therefore acknowledge both ongoing policy and programmatic efforts by HICs in each domain and the limitations of the LMIC strategies we offer as exemplars.

#### Lessons in maternal health.

The HIC challenge: Maternal health demonstrates that assumptions of HIC invulnerability are increasingly difficult to sustain as studies show that maternal mortality rates in both the U.S. and Canada compare to those in some LMICs. America’s maternal mortality rate (MMR)—18.6 death per 100,000 live-births in 2023—is the highest among HICs [48,49]. Black women have 3.5 times higher rates than white women at 50.3 deaths versus 14.5 deaths per 100,000 live births, respectively [50] and American Indian and Alaska Native women die in childbirth at double and triple the rate of white women [51,52]. Although Canada’s overall maternal mortality is low at 10–11 per 100,000 live-births, Indigenous women face an estimated twofold higher risk than the national average, with remote communities suffering much worse outcomes [53,54]. Similar disparities exist in other HICs. A study of eight countries in Europe found that, in all but one country, maternal mortality ratios were at least fifty percent higher among women born abroad or of minoritized identities [55].

These statistics show that living in an HIC does not protect one from the socio-historical factors that drive health inequities. Structural violence (defined as social institutions that harm individuals by preventing them from meeting their basic needs) [56], unequal access to care, and policy neglect contribute to poor maternal health in HICs just as they do in LMICs. Anthropologist Dana-Ain Davis [57] has argued that obstetric racism—a combination of medical racism [58] and obstetric violence [59]– exposes Black and minority women to disrespectful treatment and medical neglect and has deep historical roots that result in disproportionately high maternal mortality among racial minorities in the U.S. Similar trends exist in Canada too [60]. Provider disrespect and abuse of pregnant women have typically been studied as global health problems in LMICs [61] and less so in HICs.

The LMIC response: Many LMICs have made significant progress in maternal health despite limited resources. Sri Lanka is often highlighted as a notable success story [62] having reduced its maternal mortality rate to 18.32 deaths per 100,000 live births in 2023 down from 68 deaths per 100,000 live births in the 1980s [62]. This consistent decline over five decades is largely attributed to the establishment of a nationwide cadre of community-based public health midwives (PHMs) [63]. Each PHM is a trusted and highly trained community nurse-midwife who serves a defined local population. PHMs provide antenatal care, assist in low-risk deliveries, and link mothers to higher-level medical care when necessary. Studies show that embedding PHMs in local communities has helped overcome the social hierarchies that often make healthcare inaccessible to socially marginalized communities in Sri Lanka as PHMs make home-visits to support pregnant women and postpartum mothers [64,65]. Furthermore, the PHM eligibility criteria were reduced, which helped increase the recruitment of PHM trainees from rural areas, where 90% of PHMs are deployed [66]. This has ensured that PHMs typically work in socially and culturally familiar contexts, thereby increasing trust with communities.

Sri Lanka’s achievement should not obscure the fact that these gains are not evenly distributed across its population with ethnic minority women, particularly Tamil women from conflict affected regions, continuing to face barriers accessing maternal care [67]. Despite this, Sri Lanka’s experience is useful for demonstrating that investing in community-based midwives can deliver substantial gains in maternal health. Such community-based approaches have been shown to be responsible for maternal health successes in other LMICs [68].

Furthermore, in alignment with World Health Organization (WHO) recommendations, many LMICs have also adopted the Maternal and Perinatal Death Surveillance and Response (MPDSR) system. This framework constitutes a structured set of health system interventions aimed at systematically identifying, monitoring, and addressing maternal deaths. Over the past decade, the implementation of MPDSR across diverse LMIC contexts has been associated with measurable improvements in both access and quality of maternal health services [69]. A critical outcome of this process has been the institutionalisation of continuous data collection, systematic evaluation, and rigorous review mechanisms within primary health care. Notably, the review process is typically undertaken quarterly, thereby embedding a culture of regular accountability and evidence-informed decision-making into routine service delivery.

Lessons for HICs: Investment in community health structures is critical to improving access for marginalized populations. Expanding the role of community midwives in managing low-risk pregnancies could increase access to culturally responsive maternity care. In Canada, for instance, there has been an increase in the migrant population, yet there is little to no culturally focused maternal care available to migrant women. While the country has introduced Indigenous midwives to serve remote First Nations and Inuit communities, this initiative remains undercut by two structural barriers. First, the federal birth-evacuation policy still routinely transfers all Indigenous pregnant women living in rural or remote areas, including those with low-risk pregnancies, out of their communities to deliver in distant and unfamiliar urban hospitals [70]. Second, federal-provincial jurisdictional fragmentation leaves in-community midwifery chronically underfunded [71]. Recognition of Indigenous midwifery has thus not translated into community-based, culturally-competent maternal health care at scale, and the gap is even wider for migrant communities.

Despite overwhelming evidence that community-based, midwife-led models can improve maternal health outcomes for minoritized women [72] while increasing access to culturally responsive care [73], maternal care in many HICs remains predominantly physician-led [73]. In the U.S., some states now cover access to doula care via Medicaid or private health insurance, thereby ensuring that economically disadvantaged women can get culturally responsive care [74]. Doulas are often trained to be culturally competent and increasingly represent the racial and cultural backgrounds of those they serve [75]. However, expansion of doula services is relatively recent and has been slow: two states introduced Medicaid funded doula care in 2014 and only 14 states and DC currently offer it [76,77]. Furthermore, only 2 states currently mandate doula care in private health plans [78]. In both the U.S. and Canada, studies show that reimbursement rates for doulas are often set below a living wage and that burdensome billing and credentialing requirements deter many doulas from enrolling as providers [79].

These examples show that HIC initiatives to address maternal mortality have not fundamentally reorganized how maternal care is structured and delivered. Community-based maternity care therefore remains peripheral rather than constituting a routine function of primary healthcare. The Sri Lankan model makes visible what many HIC efforts still lack: a radically different institutional approach. What matters is not simply more midwives, but a model that proactively promotes grassroots, community-based, culturally-competent care for defined populations. The transferable lesson therefore is that maternal care should be organized around long-term relationships with communities rather than occasional contact with individual providers. In the U.S. and Canada, advancing maternal health will require ensuring that every pregnant woman has a designated community-based midwife and timely access to higher-level care when complications arise.

Another lesson for HICs is that they will need to integrate mandatory, routine surveillance and review of maternal health deaths and near misses into primary and secondary health care as per the WHO’s MPDSR. Canada for instance, does not currently operate a national MPDSR system and lacks a standardized national framework for reviewing maternal deaths and measuring disparities consistently [80]. Although Canada collects maternal mortality data, surveillance and review remain fragmented across jurisdictions and do not consistently trigger a structured system-wide learning and quality improvement. This issue is not merely a technical data gap. Without a standardised review of every maternal death, well-documented disparities generate no binding obligation to act. The MPDSR model is instructive precisely because it links surveillance to structured review and corrective action, creating an institutionalising pathway through which evidence routinely informs service improvement. Doing so, will close the accountability loop that HIC maternal health governance has largely failed to build.

#### Lessons in tuberculosis control.

The HIC challenge: The lessons from maternal health extend to other domains where HICs face persistent inequities. Tuberculosis (TB) control offers a compelling parallel. While TB is often framed as a disease of poverty concentrated in LMICs, marginalized populations within HICs continue to experience disproportionate TB burdens. In Canada, TB incidence among Inuit populations reached 188.7 cases per 100,000 in 2019, approximately 472 times that of Canadian-born, non-Indigenous individuals (0.4 per 100,000), though this declined to 135.1 per 100,000 in 2021 [81]. Among First Nations, TB incidence remains elevated at 23.4 per 100,000, significantly higher than the national average of 4.8 per 100,000 [82]. In the U.S. TB incidence is highest among Native Hawaiian or Pacific Islanders at 37.9 per 100,000 [83] and American Indian and Alaska Native populations experience TB incidence rates approximately 10 times higher than white populations [84].

In 2025, Kansas experienced one of the largest TB outbreaks in the U.S. since the 1950s, with 68 active cases identified in the community. While this was lower than the 170 TB cases recorded in Georgia’s homeless shelters in 2015–17, these numbers represent increasing TB infections in the country [85]. Furthermore, confirmed latent TB cases were in the hundreds in both Kansas and Georgia. In the U.S. and Canada, (im)migrants from high-burden settings also carry a disproportionate TB burden because latent infection acquired before migration reactivates under conditions of poverty, overcrowding, and inadequate post-arrival healthcare access [85,86].

The LMIC response: LMICs have developed innovative, community-based approaches to TB control that offer transferable models for addressing these disparities in HICs. Peru’s DOTS-Plus program, established in Lima in 1996 in collaboration with Partners in Health (PIH), demonstrated that multidrug-resistant tuberculosis (MDR-TB) could be successfully treated in community settings rather than hospitals [87]. The program achieved cure rates of over 83% among patients with highly resistant strains by employing community health workers, supervised by nurses, who provided directly observed therapy, individualized treatment regimens, proactive outreach to patients’ homes [88] and nutritional and social support. PIH has used this community-based approach, known as accompaniment, to successfully treat highly resistant TB in other LMICs, such as Rwanda, Haiti and Kazakhstan. These programs succeeded by moving care into communities and proactively providing supportive care, such as financial incentives for meals and housing and psycho-social support beyond clinical care. This social support component has consistently been shown to make TB care truly transformative where it is implemented [88]. Socioeconomic and geographic disparities in TB burden and treatment outcomes nonetheless persist in Peru, underscoring that community-based models require sustained equity-focused implementation to reach their full potential [89,90].

Lessons for HICs: The U.S. and Canada have not neglected TB disparities, and they both have active TB management programs [91,92]; however, current treatment approaches continue to rely on facility-based models of care. LMIC models, particularly home-based delivery by trusted community-based health workers, and active community-level surveillance offer alternative implementation strategies that place continuous community engagement at the center of TB care. Implementing these changes in the U.S. and Canada will require political will to revamp how TB care is traditionally provided. Efforts to introduce community-based TB care in the U.S. are relatively recent: the National Tuberculosis Coalition of America (NTCA) only developed national guidelines for community-based TB control in 2022 [93]. Many studies show limited political will to move TB care outside of health facilities despite overwhelming evidence that community settings, such as correctional facilities and homeless shelters, are key sources of TB transmission in the U.S. [87,93].

Investing in community-based models of care is essential for reaching geographically isolated and marginalized populations. In Canada’s northern communities, inadequate housing, geographical barriers to care, and historical trauma from TB sanitorium policies continue to sustain high TB transmission rates. Adapting Peru’s community health worker (CHW) model, where trusted local providers deliver treatment in patients’ homes while building on Indigenous principles of wellness and community-led care could transform TB care in HICs. Despite Canada’s 2018 commitment to eliminate TB among the Inuit by 2030, the country still lacks the comprehensive, community-based infrastructure that has proven successful in LMICs. The shortfall is instructive rather than incidental: progress toward the 2025 and 2030 targets has stalled. The LMIC models we highlighted do not merely parallel existing HIC initiatives. They identify a capability that remains underdeveloped in many HIC TB programmes, namely, community-embedded case-finding and treatment support rather than exclusive health facility-based care.

#### Community-health workers and community-based approaches.

The HIC challenge: Many of the LMIC successes we have discussed in this essay center community-based approaches and recognise the expertise of ordinary people, in the form of community health workers, rather than only that of highly trained professionals or of technological innovations. Community-based approaches are often viewed as essential only in LMICs due to healthcare provider shortages in these settings. However, the reality is that many HICs are themselves also facing profound healthcare provider shortages and are increasingly hiring these from LMICs [26,27]. CHW programs exist in HICs, but they are greatly underutilized and underexplored [94]. In the U.S., studies show that CHW programs waned in popularity in the 1990s and then experienced a resurgence in the 2010s due to increasing advocacy to address SDoH at community level [95]. The COVID-19 pandemic further revealed that a robust cadre of CHWs was urgently needed for quicker contact tracing in the early months of the outbreak and for reaching socially isolated community members [96]. While the pandemic led to more CHWs being trained in the U.S., with a current estimated total CHW population of 127,000 [96], studies show that political will to scale up CHW programs and invest in them at the federal level is largely lacking [97].

The LMIC response: Africa’s CHWs fill in critical human resource gaps and interact with community members in more personal ways that engender trust and facilitate healthcare utilization. There are innumerable examples of Africa’s CHWs providing non-judgemental psycho-social support to people who are living with HIV thus increasing HIV testing and treatment uptake [98]. Many countries in Africa have also had great success with community-based models to HIV care, which have reduced the time and monetary demands of HIV care for rural and low-income urban populations and improved access [99]. In Asia, India’s million-strong Accredited Social Health Activists (ASHAs), in turn, provide health education and basic health services in very remote rural areas and to geographically isolated populations each year and have officially been recognised by the WHO for their effectiveness in saving millions of lives over the decades [100].

We acknowledge that CHW programs in LMICs have limitations, many of which are structurally based. The ASHA program in India, for instance, has been accused of exploiting the labor of socially marginalized groups, namely women from low-income communities and who are ethnic minorities, by underpaying them despite clear evidence of their central role in India’s healthcare system [100]. CHWs in South Africa, many of whom are women, have been shown to be vulnerable to physical and sexual violence when they make home-visits [98]. Most also lack appropriate supplies, such as personal protective equipment, and face increased risk of infection, especially to communicable diseases [98].

Lessons for HICs: Canada and the U.S. could learn much from CHW programs in LMICs. These programs consistently show that community-based care models are effective at improving health outcomes in communities despite relying on CHWs who often only have basic training in health and lack college degrees. The U.S. and Canada could benefit from scaling up their CHW programs in order to reach socially isolated and marginalized populations. Several CHW Bills have recently been introduced in the U.S. Congress to lobby for federal investment in CHWs to ensure consistent and stable remuneration across states, among other concerns [101]. The barrier, however, is not simply insufficient advocacy. HIC CHW programs have repeatedly failed to scale up because their financing remains a patchwork of short-term grants and pandemic-era funds. Most states still lack stable reimbursement mechanisms, and where Medicaid reimbursement exists, rates commonly fall below the level needed to sustain a program [100]. The lesson for HICs is therefore not only a mindset shift but a financing and integration commitment that current efforts have avoided. The ASHA programme offers an important lesson to HICs because it is not a pilot project dependent on soft money, but is publicly financed and institutionalised, thus ensuring that ASHAs have a clearly defined place in the health system. HICs should scale-up CHW programmes and make them permanent fixtures of primary healthcare systems.

## Conclusion

We have argued in this essay that HICs have global health challenges of their own and could learn from global health successes experienced in LMICs. We proposed a reciprocal learning approach, which draws practical lessons from LMIC contexts where health systems have been compelled to achieve more with less. A reciprocal learning approach positions LMICs as sources of global health expertise that can help solve global health problems in HICs and decenters the hegemony of western epistemologies. Global health is about shared global vulnerabilities and requires openness to other ways of knowing and doing.

## References

[1] Koplan JP, Bond TC, Merson MH, Reddy KS, Rodriguez MH, Sewankambo NK, et al. Towards a common definition of global health. Lancet. 2009;373(9679):1993–5. doi: 10.1016/S0140-6736(09)60332-9 19493564 PMC9905260

[2] Abdalla SM, Galea S. Reimagining global health scholarship to tackle health inequities. SSM Popul Health. 2024;28:101711. doi: 10.1016/j.ssmph.2024.101711 39512546 PMC11541687

[3] Looi M-K, Dyer O. Trump watch: HIV/AIDS research sent into crisis, growing hostility to mRNA vaccines, and more. BMJ. 2025;388:r645. doi: 10.1136/bmj.r645 40164456

[4] Government of Canada. Budget 2025: A strong and sovereign Canada. Department of Finance Canada. 2024 [cited 2026 Apr 2]. Available from: https://www.budget.canada.ca/2025/home-accueil-en.html

[5] Syed SB, Dadwal V, Martin G. Reverse innovation in global health systems: towards global innovation flow. Global Health. 2013;9:36. doi: 10.1186/1744-8603-9-36 23992598 PMC3846449

[6] Zhou J, Blaylock R, Harris M. Systematic review of early abortion services in low- and middle-income country primary care: potential for reverse innovation and application in the UK context. Global Health. 2020;16(1):91. doi: 10.1186/s12992-020-00613-z 32993694 PMC7524570

[7] Arora G, Russ C, Batra M, Butteris SM, Watts J, Pitt MB. Bidirectional Exchange in Global Health: Moving Toward True Global Health Partnership. Am J Trop Med Hyg. 2017;97(1):6–9. doi: 10.4269/ajtmh.16-0982 28719333 PMC5508910

[8] Sors TG, O’Brien RC, Scanlon ML, Bermel LY, Chikowe I, Gardner A, et al. Reciprocal innovation: A new approach to equitable and mutually beneficial global health partnerships. Glob Public Health. 2023;18(1):2102202. doi: 10.1080/17441692.2022.2102202 35877989 PMC9873831

[9] Chaudhuri MM, Mkumba L, Raveendran Y, Smith RD. Decolonising global health: beyond “reformative” roadmaps and towards decolonial thought. BMJ Glob Health. 2021;6(7):e006371. doi: 10.1136/bmjgh-2021-006371 34244205 PMC8268885

[10] Mehjabeen D, Patel K, Jindal RM. Decolonizing global health: a scoping review. BMC Health Serv Res. 2025;25(1):828. doi: 10.1186/s12913-025-12890-8 40598071 PMC12211805

[11] Affun-Adegbulu C, Adegbulu O. Decolonising Global (Public) Health: from Western universalism to Global pluriversalities. BMJ Glob Health. 2020;5(8):e002947. doi: 10.1136/bmjgh-2020-002947 32819916 PMC7443258

[12] Jackon P, Henry C. The needs of the ‘other’ global health: The case of Remote Area Medical. In: Herrick C, Reubi D, editors. Global health and geographical imaginaries. Routledge. 2017.

[13] Masvawure TB. Global health as analyticMaking sense of the domestic COVID responses in the United States. The Routledge Handbook of Anthropology and Global Health. Routledge. 2024. 322–38. doi: 10.4324/9781003284345-28

[14] Nouvet E, Ndu M, Pratt B, Arguedas Ramirez G, Prainsack B, Karunakara U, et al. Meanings and practices of solidarity in global health: a qualitative investigation - study protocol. BMJ Open. 2026;16(1):e095243. doi: 10.1136/bmjopen-2024-095243 41500618 PMC12781975

[15] Mason PH, Kerridge I, Lipworth W. The Global in Global Health is Not a Given. Am J Trop Med Hyg. 2017;96(4):767–9. doi: 10.4269/ajtmh.16-0791 28138044 PMC5392617

[16] Congressional Research Service. U.S. Agency for International Development: An Overview. 2025 [cited 2026 Apr 2]. Available from: https://www.congress.gov/crs-product/IF10261

[17] Association of American Universities. Federal Research Cuts Threaten U.S. Innovation and Leadership. 2025 [cited 2026 April 2]. Available from: https://www.aau.edu/key-issues/federal-research-cuts-threaten-us-innovation-and-leadership

[18] USAID Stop-Work. Tracking. Informing. 2026 [cited 2026 January 20]. Available from: https://www.usaidstopwork.com

[19] Bandara S, Fieldhouse JK, Alwis I, Abascal Miguel L, Christian C, Evaborhene N. Disinvesting in the future leadership of global health has already begun: What can we do about it?. PLOS Glob Public Health. 2025;5(10):e0005310. doi: 10.1371/journal.pgph.0005310 41160591 PMC12571303

[20] U.S. Department of State. America First Global Health Strategy. 2025 [cited March 19 2026]. Available from: https://www.state.gov/wp-content/uploads/2025/09/America-First-Global-Health-Strategy-Report.pdf

[21] Government of Canada N. Our north, strong and free: A renewed vision for Canada’s defence. 2024 [cited 2026 April 2]. Available from: https://www.canada.ca/en/department-national-defence/corporate/reports-publications/north-strong-free-2024.html

[22] Sassmannshausen F. Global Fund faces $5bn shortfall as France slashes support, EU delays pledge. Health Policy Watch. 2026 [cited 2026 March 10]. Available from: https://healthpolicy-watch.news/global-fund-shortfall/

[23] OECD. Cuts in official development assistance: OECD projections for 2025 and the near term. Paris: OECD Publishing. 2025. [cited June 30 2026]. doi: 10.1787/8c530629-en

[24] Mirugwe A. The cost of blind spots: How the 2025 USAID closure weakened global outbreak detection. 2026 [cited 2026 March 1]. https://ssrn.com/abstract=6797738 10.3389/fpubh.2026.1887795 PMC13581717 42755672

[25] Schreiber M. US is ‘simply choosing not to stop’ Ebola outbreak after massive public health cuts, experts say. The Guardian. 2026 [citex 2026 June 4]. https://www.theguardian.com/world/2026/may/21/ebola-outbreak-public-health

[26] Sweetman A. 3 The Migration of Health Professionals to Canada: Reducing Brain Waste and Improving Labour Market Integration. Global Migration, Gender, and Health Professional Credentials. University of Toronto Press. 2022. p. 69–94. doi: 10.3138/9781487531744-006

[27] Health Canada. Characteristics and labour market outcomes of internationally educated health care professionals in Canada. 2023 [cited 2026 April 2]. Available from: https://www.canada.ca/en/health-canada/services/health-care-system/health-human-resources/characteristics-labour-market-outcomes-internationally-educated-health-care-professionals-canada.html

[28] Alemu K, Berhanu D, Bergström A, Hoa DP, Swartling Peterson S, Phoummalaysith B, et al. Towards equitable partnerships in global health research: experiences from Ethiopia, Uganda, Lao PDR and Vietnam. BMJ Glob Health. 2025;10(6):e019130. doi: 10.1136/bmjgh-2025-019130 40588294 PMC12211826

[29] Abimbola S, Asthana S, Montenegro C, Guinto RR, Jumbam DT, Louskieter L, et al. Addressing power asymmetries in global health: Imperatives in the wake of the COVID-19 pandemic. PLoS Med. 2021;18(4):e1003604. doi: 10.1371/journal.pmed.1003604 33886540 PMC8101997

[30] Bourgois P. The moral economies of homeless heroin addicts: confronting ethnography, HIV risk, and everyday violence in San Francisco shooting encampments. Substance Use & Misuse. 1998;33(11):2323–53. 9758016 10.3109/10826089809056260

[31] Fadiman A. The spirit catches you and you fall down. Farrax, Straus and Giroux. 1997.

[32] Marmot M. Social determinants of health inequalities. Lancet. 2005;365(9464):1099–104. doi: 10.1016/S0140-6736(05)71146-6 15781105

[33] Macias-Konstantopoulos WL, Collins KA, Diaz R, Duber HC, Edwards CD, Hsu AP, et al. Race, Healthcare, and Health Disparities: A Critical Review and Recommendations for Advancing Health Equity. West J Emerg Med. 2023;24(5):906–18. doi: 10.5811/westjem.58408 37788031 PMC10527840

[34] Srugo SA, Ricci C, Leason J, Jiang Y, Luo W, Nelson C, et al. Disparities in primary and emergency health care among “off-reserve” Indigenous females compared with non-Indigenous females aged 15-55 years in Canada. CMAJ. 2023;195(33):E1097–111. doi: 10.1503/cmaj.221407 37640405 PMC10462408

[35] Galea S. The contagion next time. Oxford: Oxford University Press. 2021.

[36] Kluender R, Mahoney N, Wong F, Yin W. Medical debt in the US, 2009-2020. JAMA. 2021;326(3):250–6. doi: 10.1001/jama.2021.8694 34283184 PMC8293024

[37] Government of Canada, Indigenous Services Canada. Active long-term drinking water advisories. 2021 [cited 2026 January 19]. Available from: https://www.sac-isc.gc.ca/eng/1614387410146/1614387435325

[38] Maddox R, Funnell S. Our Health Counts: Nothing about us without us in our right to be counted. Can J Public Health. 2024;115(Suppl 2):187–92. doi: 10.17269/s41997-024-00928-z 39115790 PMC11582229

[39] Galabuzi GE. Social Exclusion. In: Raphael D, editor. Social determinants of health: Canadian perspectives. 3rd ed. Canadian Scholars’ Press; 2016. 353–66.

[40] Pottie K, Greenaway C, Feightner J, Welch V, Swinkels H, Rashid M, et al. Evidence-based clinical guidelines for immigrants and refugees. CMAJ. 2011;183(12):E824-925. doi: 10.1503/cmaj.090313 20530168 PMC3168666

[41] Larson HJ, Jarrett C, Eckersberger E, Smith DMD, Paterson P. Understanding vaccine hesitancy around vaccines and vaccination from a global perspective: a systematic review of published literature, 2007-2012. Vaccine. 2014;32(19):2150–9. doi: 10.1016/j.vaccine.2014.01.081 24598724

[42] Feemster KA, Szipszky C. Resurgence of measles in the United States: how did we get here?. Curr Opin Pediatr. 2020;32(1):139–44. doi: 10.1097/MOP.0000000000000845 31790030

[43] Crosby AW. The Columbian exchange: biological and cultural consequences of 1492. Bloomsbury Publishing USA. 2003.

[44] Fassin D. When bodies remember: Experiences and politics of AIDS in South Africa. California: California University Press. 2007.

[45] Mavodza C, Goldman R, Cooper B. The impacts of the global gag rule on global health: a scoping review. Glob Health Res Policy. 2019;4:26. doi: 10.1186/s41256-019-0113-3 31485484 PMC6714436

[46] OECD. Development co‑operation profiles. OECD. 2024 [cited 2026 April 2]. Available from: https://www.oecd.org/en/publications/development-co-operation-profiles_2dcf1367-en.html

[47] Escobar A. Designs for the pluriverse: Radical interdependence, autonomy, and the making of worlds. Durham: Duke University Press. 2018.

[48] Hoyert DL. Maternal Mortality Rates in the United States, 2022. Centers for Disease Control and Prevention (U.S.). 2024 [cited 2026 April 2]. Available from: https://stacks.cdc.gov

[49] Joseph KS, Lisonkova S, Boutin A, Muraca GM, Razaz N, John S, et al. Maternal mortality in the United States: are the high and rising rates due to changes in obstetrical factors, maternal medical conditions, or maternal mortality surveillance?. Am J Obstet Gynecol. 2024;230(4):440.e1-440.e13. doi: 10.1016/j.ajog.2023.12.038 38480029

[50] MacDorman MF, Thomas M, Declercq E, Howell EA. Racial and ethnic disparities in maternal mortality in the United States using enhanced vital records, 2016‒2017. Am J Public Health. 2021;111(9):1673–81. doi: 10.2105/AJPH.2021.306375 34383557 PMC8563010

[51] Patterson EJ, Becker A, Baluran DA. Gendered Racism on the Body: An Intersectional Approach to Maternal Mortality in the United States. Popul Res Policy Rev. 2022;41(3):1261–94. doi: 10.1007/s11113-021-09691-2

[52] Kozhimannil KB, Interrante JD, Tofte AN, Admon LK. Severe Maternal Morbidity and Mortality Among Indigenous Women in the United States. Obstet Gynecol. 2020;135(2):294–300. doi: 10.1097/AOG.0000000000003647 31923072 PMC7012336

[53] Weckman AM, Farrugia P. Inequities in Canadian maternal-child healthcare are perpetuating the intergenerational effects of colonization for indigenous women and children. Front Glob Womens Health. 2025;6:1513145. doi: 10.3389/fgwh.2025.1513145 40535705 PMC12174450

[54] Maxwell C, Tunde-Byass M, Wilson-Mitchell K. Achieving equity in reproductive care and birth outcomes for Black people in Canada. CMAJ. 2024;196(10):E343–5. doi: 10.1503/cmaj.231105 38499307 PMC10948181

[55] Diguisto C, Saucedo M, Kallianidis A, Bloemenkamp K, Bødker B, Buoncristiano M, et al. Maternal mortality in eight European countries with enhanced surveillance systems: descriptive population based study. BMJ. 2022;379:e070621. doi: 10.1136/bmj-2022-070621 36384872 PMC9667469

[56] Farmer PE, Nizeye B, Stulac S, Keshavjee S. Structural violence and clinical medicine. PLoS Med. 2006;3(10):e449. doi: 10.1371/journal.pmed.0030449 17076568 PMC1621099

[57] Davis D-A. Obstetric Racism: The Racial Politics of Pregnancy, Labor, and Birthing. Med Anthropol. 2019;38(7):560–73. doi: 10.1080/01459740.2018.1549389 30521376

[58] Washington HA. Medical apartheid: The dark history of medical experimentation on Black Americans from colonial times to the present. Doubleday Books. 2006.

[59] O’Brien E, Rich M. Obstetric violence in historical perspective. Lancet. 2022;399(10342):2183–5. doi: 10.1016/S0140-6736(22)01022-4 35691313

[60] El-Mowafi IM, Yalahow A, Idriss-Wheeler D, Yaya S. The politest form of racism: sexual and reproductive health and rights paradigm in Canada. Reprod Health. 2021;18(1):59. doi: 10.1186/s12978-021-01117-8 33750408 PMC7940862

[61] Bohren MA, Hunter EC, Munthe-Kaas HM, Souza JP, Vogel JP, Gülmezoglu AM. Facilitators and barriers to facility-based delivery in low- and middle-income countries: a qualitative evidence synthesis. Reprod Health. 2014;11(1):71. doi: 10.1186/1742-4755-11-71 25238684 PMC4247708

[62] WHO. WHO Data. World Health Organization. 2025 [citd 2025 October 2]. Available from: https://data.who.int/countries/144

[63] Senanayake L, Ranathunga A, Kaluarachchi A. Maternal Health in Sri Lanka: 75 years of national commitment towards excellence. Ceylon Med J. 2023;68(S1):46–52. doi: 10.4038/cmj.v68isi1.9767 37610968

[64] S1S45Haththotuwa R, Senanayake L, Senarath U, Attygalle D. Models of care that have reduced maternal mortality and morbidity in Sri Lanka. Int J Gynaecol Obstet. 2012;119 Suppl 1:S45-9. doi: 10.1016/j.ijgo.2012.03.016 22883911

[65] Udayanga S, De Zoysa LS, Bellanthudawa A. Mobilising Communities Prior to Healthcare Interventions: Reflections on the Role of Public Health Midwives Working With Vulnerable Communities of Sri Lanka. Community Health Equity Res Policy. 2024;45(1):2752535X241232000. doi: 10.1177/2752535X241232000 38308494 PMC11418316

[66] Karunathilake I, De Silva P. Retention of family healthworkers in rural communities as an important strategy in task-shifting-the Sri Lankan experience. JMAJ. 2010;53(6):424–30.

[67] Kottegoda S, Samuel K, Emmanuel S. Reproductive health concerns in six conflict-affected areas of Sri Lanka. Reprod Health Matters. 2008;16(31):75–82. doi: 10.1016/S0968-8080(08)31359-7 18513609

[68] Exemplars in Global Health. [Accessed 2026 July 15]. https://www.exemplars.health

[69] Russell N, Tappis H, Mwanga JP, Black B, Thapa K, Handzel E, et al. Implementation of maternal and perinatal death surveillance and response (MPDSR) in humanitarian settings: insights and experiences of humanitarian health practitioners and global technical expert meeting attendees. Confl Health. 2022;16(1):23. doi: 10.1186/s13031-022-00440-6 35526012 PMC9077967

[70] Campbell E, Murdock M, Durant S, Couchie C, Meekis C, Rae C, et al. Indigenous Peoples’ responses to evacuation for birth in Ontario: conceptualizing risk through an Indigenous midwifery-led approach. Int J Equity Health. 2025;24(1):135. 40361221 10.1186/s12939-025-02491-6 PMC12070555

[71] Wodtke L, Hayward A, Nychuk A, Doenmez C, Sinclair S, Cidro J. The need for sustainable funding for Indigenous doula services in Canada. Womens Health (Lond). 2022;18:17455057221093928. doi: 10.1177/17455057221093928 35438029 PMC9021521

[72] Scroggins JK, Harkins SE, Brown S, St Clair V, LeBron GK, Barcelona V. A systematic review of community-based interventions to address perinatal mental health. Semin Perinatol. 2024;48(6):151945. doi: 10.1016/j.semperi.2024.151945 39033052 PMC11377151

[73] McLachlan HL, Newton M, McLardie-Hore FE, McCalman P, Jackomos M, Bundle G, et al. Translating evidence into practice: Implementing culturally safe continuity of midwifery care for First Nations women in three maternity services in Victoria, Australia. EClinicalMedicine. 2022;47:101415. doi: 10.1016/j.eclinm.2022.101415 35747161 PMC9142789

[74] Sandall J, Fernandez Turienzo C, Devane D, Soltani H, Gillespie P, Gates S, et al. Midwife continuity of care models versus other models of care for childbearing women. Cochrane Database Syst Rev. 2024;4(4):CD004667. doi: 10.1002/14651858.CD004667.pub6 38597126 PMC11005019

[75] Louis-Jacques AF, Applequist J, Perkins M, Williams C, Joglekar R, Powis R, et al. Florida Doulas’ Perspectives on Their Role in Reducing Maternal Morbidity and Health Disparities. Womens Health Issues. 2024;34(4):417–28. doi: 10.1016/j.whi.2024.01.003 38503681

[76] Mass.gov. MassHealth Doula Services Program: Information for MassHealth Members. 2024 [cited 2026 April 2]. Available from: https://www.mass.gov/info-details/masshealth-doula-services-program-information-for-masshealth-members

[77] Falconi AM, Ramirez L, Cobb R, Levin C, Nguyen M, Inglis T. Role of doulas in improving maternal health and health equity among Medicaid enrollees, 2014-2023. Am J Pub Health. 2024;114(11):1275–85. doi: 10.2105/AJPH.2024.307805 39356988 PMC11447808

[78] Herbert K. Doula care: Spring 2025 state of the states. National Health Law Program. [Accessed 2026 July 20]. https://healthlaw.org/private-insurance-coverage-of-doula-care-spring-2024-state-of-the-states/

[79] Medicaid and CHIP Payment and Access Commission (MACPAC). Doulas in Medicaid: Findings from a case study. Washington (DC): MACPAC. 2023. https://www.macpac.gov/publication/doulas-in-medicaid-case-study-findings/

[80] Murphy-Kaulbeck L. SOGC Calls for National Framework for Maternal Mortality Data Collection. Media Release. 2024 [cited 2026 March 21]. Available from: https://sogc.org/en/en/content/featured-news/SOGC-Calls-for-National-Framework-for-Maternal-Mortality-Data-Collection.aspx

[81] Public Health Agency of Tuberculosis in Canada: 2012 to 2021 Expanded Report. Government of Canada. 2023 [cited 2026 Apr 2]. Available from: https://www.canada.ca/en/public-health/services/publications/diseases-conditions/tuberculosis-canada-expanded-report-2012-2021.html

[82] Jetty R. Tuberculosis among First Nations, Inuit and Métis children and youth in Canada: Beyond medical management. Paediatr Child Health. 2020;26(2):e78–81. doi: 10.1093/pch/pxz183 33747314 PMC7962700

[83] CDC. Tuberculosis in the United States, 2024. Executive Commentary. 2025 [cited 2026 April 2]. Available from: https://www.cdc.gov/tb-surveillance-report-2024/executive-commentary/index.html

[84] Springer YP, Kammerer JS, Silk BJ, Langer AJ. Tuberculosis in Indigenous Persons - United States, 2009-2019. J Racial Ethn Health Disparities. 2022;9(5):1750–64. doi: 10.1007/s40615-021-01112-6 34448124 PMC8881557

[85] American Association of Immunologists. What we know about the Tuberculosis outbreak in Kansas. 2025 [Accessed 2026 June 30]. https://news.aai.org/2025/02/05/tuberculosis-outbreak-kansas/

[86] Yanga N. Advocating for Change: Addressing Barriers To Tuberculosis Care for Immigrants and Refugees in Canada. J Immigr Minor Health. 2025;27(5):647–51. doi: 10.1007/s10903-025-01744-4 40748445

[87] Mitnick C, Bayona J, Palacios E, Shin S, Furin J, Alcántara F, et al. Community-based therapy for multidrug-resistant tuberculosis in Lima, Peru. N Engl J Med. 2003;348(2):119–28. doi: 10.1056/NEJMoa022928 12519922

[88] Shin S, Furin J, Bayona J, Mate K, Kim JY, Farmer P. Community-based treatment of multidrug-resistant tuberculosis in Lima, Peru: 7 years of experience. Soc Sci Med. 2004;59(7):1529–39. doi: 10.1016/j.socscimed.2004.01.027 15246180

[89] Saunders MJ, Montoya R, Quevedo L, Ramos E, Datta S, Evans CA. The social determinants of tuberculosis: a case-control study characterising pathways to equitable intervention in Peru. Infect Dis Poverty. 2025;14(1):53. doi: 10.1186/s40249-025-01324-6 40537847 PMC12180230

[90] Asabor EN, Vermund SH. Confronting Structural Racism in the Prevention and Control of Tuberculosis in the United States. Clin Infect Dis. 2021;73(9):e3531–5. doi: 10.1093/cid/ciaa1763 33242078 PMC8563219

[91] Public Health Agency of Tuberculosis in Canada: 2012 to 2021 Expanded Report. Government of Canada. 2023 [cited 2026 Apr 2]. Available from: https://www.canada.ca/en/public-health/services/publications/diseases-conditions/tuberculosis-canada-expanded-report-2012-2021.html

[92] Rana N, Johnston JC, Schwartzman K, Oxlade O, Suarez PG, Gasana M, et al. Achieving tuberculosis elimination in Canada and the USA: giving equal weight to domestic and international efforts. BMC Global Public Health. 2024;2(1). doi: 10.1186/s44263-024-00115-9

[93] ShahM, DanskyZ, NathavitharanaR, BehmH, BrownS, DovL, et al. Guidelines for respiratory isolation and restrictions to reduce transmission of pulmonary tuberculosis in community settings. Clinical Infectious Diseases. 2024. doi: 10.1093/cid/ciae19938632829

[94] Cherrington A, Ayala GX, Elder JP, Arredondo EM, Fouad M, Scarinci I. Recognizing the diverse roles of community health workers in the elimination of health disparities: from paid staff to volunteers. Ethn Dis. 2010;20(2):189–94. 20503902 PMC3695477

[95] Rodriguez NM. Community Health Workers in the United States: Time to Expand a Critical Workforce. Am J Public Health. 2022;112(5):697–9. doi: 10.2105/AJPH.2022.306775 35324266 PMC9010926

[96] Peretz PJ, Islam N, Matiz LA. Community health workers and covid-19: addressing social determinants of health in times of crisis and beyond. N Engl J Med. 2020;383(19):e108. doi: 10.1056/NEJMp202264 32966715

[97] Schmit CD, Washburn DJ, LaFleur M, Martinez D, Thompson E, Callaghan T. Community Health Worker Sustainability: Funding, Payment, and Reimbursement Laws in the United States. Public Health Rep. 2022;137(3):597–603. doi: 10.1177/00333549211006072 33909522 PMC9109543

[98] Mantell JE, Masvawure TB, Zech JM, Reidy W, Msukwa M, Glenshaw M, et al. “They are our eyes outside there in the community”: Implementing enhanced training, management and monitoring of South Africa’s ward-based primary healthcare outreach teams. PLoS One. 2022;17(8):e0266445. doi: 10.1371/journal.pone.0266445 36018854 PMC9417004

[99] Bochner AF, Meacham E, Mhungu N, Manyanga P, Petracca F, Muserere C, et al. The rollout of Community ART Refill Groups in Zimbabwe: a qualitative evaluation. J Int AIDS Soc. 2019;22(8):e25393. doi: 10.1002/jia2.25393 31454178 PMC6711352

[100] Pandhi N. The ‘caste’ of decolonization: Structural casteism, public health praxis, and radical accountability in contemporary India. In: Masvawure TB, Foley EE, editors. The Routledge Handbook of Anthropology and Global Health. Routledge. 2024. 19–34.

[101] Basu S, Patel SY, Robinson K, Baum A. Financing Thresholds for Sustainability of Community Health Worker Programs for Patients Receiving Medicaid Across the United States. J Community Health. 2024;49(4):606–34. doi: 10.1007/s10900-023-01290-w 38311699 PMC11306546

